# Dramatic regression of macular and peripheral retinoschisis with dorzolamide 2 % in X-linked retinoschisis: a case report

**DOI:** 10.1186/s13256-016-0905-8

**Published:** 2016-06-01

**Authors:** Ama Sadaka, Robert A. Sisk

**Affiliations:** 1 Department of Ophthalmology, University of Cincinnati College of Medicine, 231 Albert Sabin Way, Cincinnati, OH 45267 USA; 2 Cincinnati Children’s Hospital Medical Center, Cincinnati, OH USA; 3 Cincinnati Eye Institute, Cincinnati, OH USA

**Keywords:** Dorzolamide, TRUSOPT, Carbonic anhydrase inhibitors, X-linked retinoschisis, Retinal detachment

## Abstract

**Background:**

X-linked retinoschisis is one of the more frequently encountered inherited macular retinal disorders affecting young males, causing loss of vision. Patients exhibit macular schisis and peripheral schisis, which can mimic retinal detachment, a very different entity that requires surgical intervention.

**Case presentation:**

An 8-month-old African-American boy was presented to our hospital with severe X-linked retinoschisis involving symmetrical bullous peripheral retinoschisis in both eyes, mimicking retinal detachment. One eye received multiple surgeries for retinal detachment repair that were complicated by proliferative vitreoretinopathy. Later, portable optical coherence tomography was used to confirm absence of retinal detachment despite a corrugated fundus appearance in the fellow eye. Following treatment with topical dorzolamide 2 % for 18 months, there was dramatic regression of both macular and peripheral schisis cavities in the nonoperative eye.

**Conclusions:**

Severe bullous peripheral schisis in infants with severe X-linked retinoschisis may produce posterior corrugations that mimic rhegmatogenous retinal detachment. Clinical suspicion for retinal detachment in infants with X-linked retinoschisis should be confirmed by portable optical coherence tomography before surgical intervention. Bullous peripheral schisis can remain clinically stable over time, but topical dorzolamide 2 % may facilitate collapse.

## Background

X-linked retinoschisis (XLRS), caused by a mutation in the retinoschisin gene (*RS1*) [1], has been reported among multiple ethnicities and has an estimated prevalence of between 1 in 15,000 and 1 in 30,000 people [2]. Almost all patients exhibit macular schisis, and half have peripheral schisis. Optical coherence tomography (OCT) demonstrates that macular schisis involves all retinal layers, while extramacular schisis occurs near the nerve fiber layer [1, 3]. Persistence of macular schisis eventually degrades into macular atrophy and permanent loss of vision. Peripheral involvement can be complicated by vitreous hemorrhage and retinal detachment (RD). Schisis can be severe, mimicking RD; however, treatment for these two entities is very different.

Carbonic anhydrase inhibitors (CAIs) have been used for treatment of macular schisis of various causes [3]. CAIs function by acidifying the subretinal space, increasing fluid transport across the retinal pigment epithelium. Although CAIs have been reported to reduce mild midperipheral schisis to a degree measurable by OCT [4], we present a case of a patient with XLRS, which was thought to be RD on initial examination, who had a dramatic response of both macular and severe peripheral schisis to topical dorzolamide 2 %.

## Case presentation

**Fig. 1:**
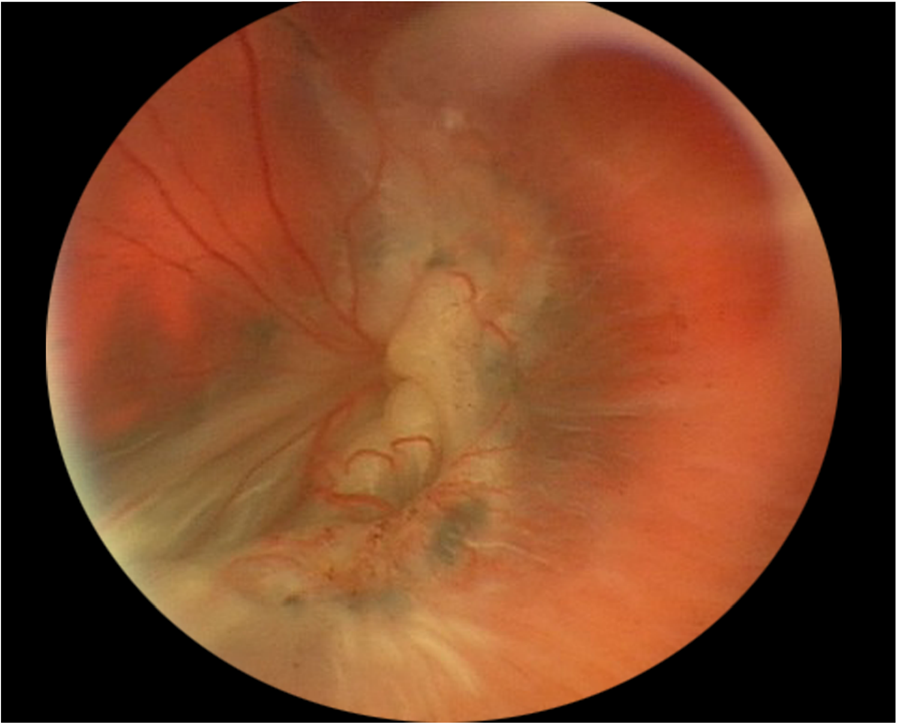
Fundus photograph of the left eye showing total retinal detachment with extensive proliferative vitreoretinopathy after multiple surgeries

An 8-month-old otherwise healthy African-American boy was presented to our hospital with poor visual behavior. His past medical history was unremarkable, and he had no contributory toxic or medication exposures. His visual acuity at presentation was measured as centered, steady but unmaintained in both eyes, and with a negative induced tropia test. His pupillary responses were sluggish, but he had no afferent pupillary defect. His examination showed very bullous anterior retinal elevations, posterior retinal corrugations, and cystic foveae that appeared to represent severe XLRS with bilateral chronic rhegmatogenous RDs and macular holes (MHs) (Fig. 1). Genetic testing revealed a hemizygous R197H mutation in the *RS1* gene.

**Fig. 2:**
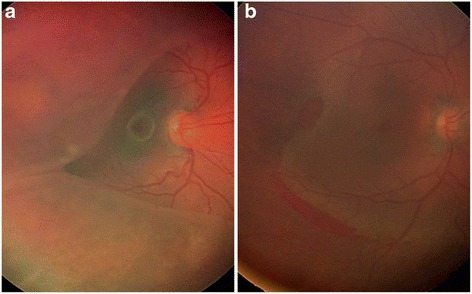
Fundus photographs of the patient’s right eye. **a** Posterior corrugation and cystic fovea appearing like retinal detachment before treatment. **b** Regression of macular and peripheral schisis cavities following topical dorzolamide with residual intraschisis hemorrhage

**Fig. 3:**
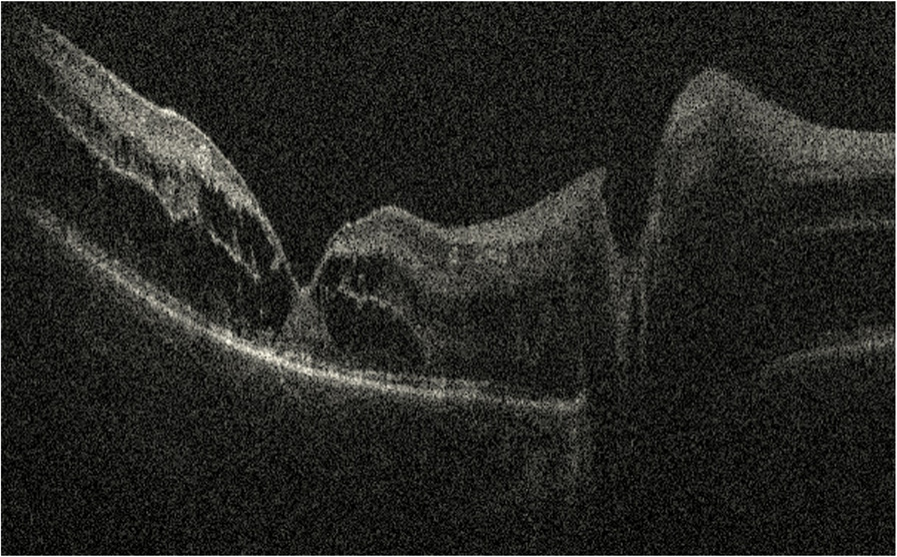
Optical coherence tomographic image of the patient’s right eye after treatment with dorzolamide, showing termination of cyst walls with deep foveal depression

The patient underwent unsuccessful schisis drainage externally, followed by vitrectomy for RD repair. The repair was complicated by multiple recurrences of RD from proliferative vitreoretinopathy (PVR) that ultimately resolved following extensive retinectomy and silicone oil tamponade. The patient’s right eye (Fig. 2a) was started on dorzolamide 2 % twice per day. Following 18 months of therapy, a dramatic regression of both macular and peripheral schisis cavities with residual intraschisis hemorrhage were seen by portable OCT during examination with the patient under anesthesia (Fig. 3). Rather than the foveal convexity and central cyst typically observed in typical XLRS, OCT of the right eye showed an abnormally deep foveal depression with termination of cyst walls at the vertical midline of the fovea, consistent with resolved MH and resolution of severe macular and peripheral retinoschisis (Figs. 2b and 3) [5]. Partial vitreous separation accompanied peripheral schisis resolution. The boy’s visual acuity at his last visit was measured as 20/150 in the right eye using the LEA Symbols Test and no light perception on the left, which had undergone surgery. At the boy’s last follow-up visit, reduced compliance with topical dorzolamide 2 % had resulted in increased macular schisis, so the medication will be continued indefinitely to prevent schisis-related anatomic and visual complications.

## Discussion

XLRS, first described in 1898, is one of the more frequently encountered inherited macular retinal disorders affecting young males [6]. Histologic studies have shown a split and/or schisis within the superficial retinal layers, the inner limiting membrane, the nerve fiber layer, and the ganglion cell layer. The ganglion cell layer was found to be thinned, with marked degeneration of photoreceptors associated with thinning of the inner nuclear layer [2].

Vitreous hemorrhages and RD, which remain major complications, can occur at any age. Full-thickness MHs have rarely been reported [7]. As RD becomes chronic, the retina becomes transparent and corrugations flatten, mimicking the appearance of retinoschisis. Although the presence of large outer retinal holes or demarcation lines, ultrasonographic characteristics, and response to laser photocoagulation may facilitate distinguishing between RD and retinoschisis, these features are not universal or conclusive, especially in eyes with XLRS. We assert that spectral domain optical coherence tomography (SD-OCT) is invaluable in making this distinction. The raster scan should include the margin of the retinal elevation to show whether the outer retina is separated from the RPE. Unfortunately, we could not perform imaging in the office with a conventional upright SD-OCT unit using the “flying baby” technique, and our facility did not have access to a portable SD-OCT unit at the time of initial presentation.

Retinoschisin protein has been identified throughout all neuroretinal layers and is believed to function in cell adhesion in the development and maintenance of retinal architecture, although the specific molecular interactions remain unclear [8]. However, it is unclear whether the role of retinoschisin is purely structural or has functional implications in fluid transport across the retina and RPE [8].

Carbonic anhydrase, one of the most ubiquitous enzyme systems in the body, is also found in the red-green cones, within the Mueller cells of the retina, and in the retinal pigment epithelium. CAIs have been used in ophthalmology for many years to reduce intraocular pressure. Recently, their use has been expanded to treatment of cystoid macular edema and schisis in patients with several retinal diseases, such as retinitis pigmentosa and uveitis [9]. CAIs are thought to control and adjust the extracellular pH gradients produced by the metabolic activity of cells. The administration of CAIs has been shown to enhance the fluid transporter present in the RPE barrier, as well as to improve retinal adhesiveness [10]. Improving the effectiveness of the RPE pump in eyes with XLRS has been demonstrated clinically to significantly reduce macular schisis and modestly reduce midperipheral retinoschisis [4, 11]. However, the reduction in schisis in our patient receiving topical dorzolamide 2 % was dramatic. Schisis cavities may fluctuate significantly in XLRS, but the degree of resolution in our patient is unprecedented.

The early onset of bullous schisis cavities in our patient was an indication of early and severe vitreous degeneration (syneresis). Intraoperatively, the posterior hyaloid face of the operative eye was surprisingly easy to lift off the macula, although it was more tightly adherent in areas of bullous schisis, which resulted in enlargement of inner retinal holes and required excision of the schisis cavities. This may suggest a differential expression of the functional defect associated with *RS1* mutation. We previously reported a complicated course when repairing RD in another infant with severely bullous XLRS due to an inability to separate the posterior hyaloid face from the most bullous portions of peripheral retinoschisis [12]. The vitreous remnant became a scaffold for PVR, which led to multiple recurrences of RD. Another report in the literature describes the use of combined external drainage and vitrectomy for severe XLRS; however, that also can cause outer retinal holes, increasing risk of PVR [13]. In the nonsurgical eye, it is unclear whether the partial vitreous separation was spontaneous or the result of retraction of the inner retina from schisis reduction following dorzolamide [14]. If the vitreous separation was important in schisis reduction, intravitreal autologous plasmin or ocriplasmin could be beneficial to either obviate vitrectomy or reduce the vitreoretinal scaffold for PVR formation in these complex eyes.

## Conclusions

This case report highlights several points. XLRS should always be included in the differential diagnosis for bilateral poor visual behavior in an infant boy. Severe peripheral bullous retinoschisis may create posterior corrugations that mimic rhegmatogenous RD. Clinical suspicion for RD in infants with XLRS should be confirmed preoperatively by OCT. Although vitreous separation plays an important role in resolution of peripheral bullous retinoschisis, topical dorzolamide may facilitate resolution of both severe macular and peripheral schisis.
